# Innovation Inspired by Nature: Applications of Biomimicry in Engineering Design

**DOI:** 10.3390/biomimetics9090523

**Published:** 2024-08-30

**Authors:** Teresa Aguilar-Planet, Estela Peralta

**Affiliations:** Department of Engineering Design, University of Seville, C/Virgen de África 7, 41011 Seville, Spain; maguilar8@us.es

**Keywords:** biomimicry, innovation, bioinspiration, biologically inspired method, industrial design, sustainability

## Abstract

Sustainable development is increasingly driving the trend toward the application of biomimicry as a strategy to generate environmentally friendly solutions in the design of industrial products. Nature-inspired design can contribute to the achievement of the Sustainable Development Goals by improving efficiency and minimizing the environmental impact of each design. This research conducted an analysis of available biomimetic knowledge, highlighting the most applied tools and methodologies in each industrial sector. The primary objective was to identify sectors that have experienced greater adoption of biomimicry and those where its application is still in its early stages. Additionally, by applying the available procedures and tools to a selected case study (technologies in marine environments), the advantages and challenges of the methodologies and procedures were determined, along with potential gaps and future research directions necessary for widespread implementation of biomimetics in the industry. These results provide a comprehensive approach to biomimicry applied to more sustainable practices in product design and development.

## 1. Introduction

The ever increasing need to develop sustainable products lies primarily in the ability to mitigate environmental and social impacts throughout their entire life cycle. From the initial stage of raw material extraction to the recycling or final disposal of the product, incorrect solutions can negatively affect the planet through global warming, acidification, or destruction of the ozone layer. In addition, some stages of the life cycle, such as manufacturing, may involve precarious labor conditions or exploitation. To address these issues, the Sustainable Development Goals (SDGs) [1] propose different methods and strategies. From an environmental point of view, notable goals include SDG 6 ‘Clean Water and Sanitation’, SDG 7 ‘Affordable and Clean Energy’, SDG 13 ‘Climate Action’ and SDG 15 ‘Life on Land’. From a social point of view, SDG 11 ‘Sustainable Cities and Communities’, SDG 9 ‘Industry, Innovation, and Infrastructure’, and SDG 12 ‘Responsible Consumption and Production’ promote sustainable usage patterns, reduction of the ecological footprint, improvement of labor conditions, and production of more environmentally friendly and innovative products. These goals work together to drive the development of more sustainable and ethical products and processes.

Human beings are an intrinsic component of the planet and are a part of an extensive ecosystem. Isolated actions overlook the relationship with the surrounding environment. Numerous contemporary problems, such as climate change, ocean pollution, and species extinction, among other environmental impacts, could have been prevented through proper integration with the environment. The SDGs represent an integrated approach to protecting the planet while ensuring the prosperity of the ecosystem. Successfully achieving these goals requires innovative approaches that facilitate a shift in perspective toward solving real challenges with zero environmental impact. In this context, the SDGs are fostering an emerging trend in design to create materials and technologies that not only aim to achieve sustainability through low environmental and social impact, but also possess regenerative and restorative capabilities [2]. Biomimicry can become a fundamental discipline in this setting to facilitate the R&D+i process. Its methodologies and tools provide the design team with a source of inspiration to develop sustainable solutions based on nature, using biological principles in the design and development stages of products.

**Figure 1:**
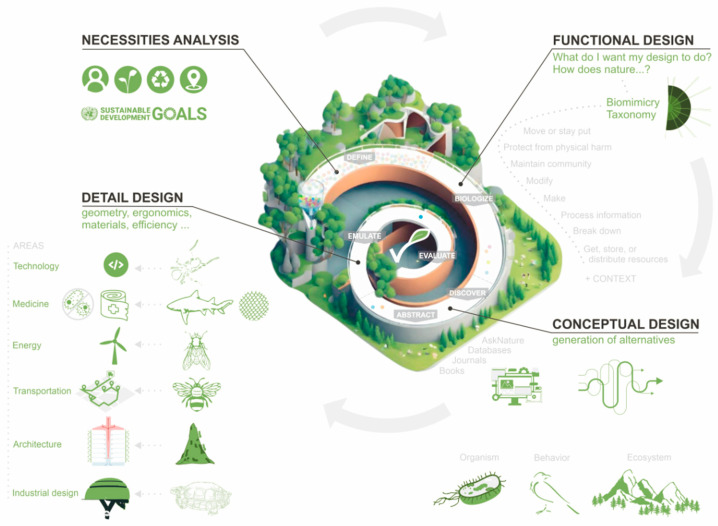
Potential application of bioinspired items in product design.

Biomimicry [3] is a discipline based on the imitation of nature to develop innovative solutions to real challenges. Its source of inspiration lies in the observation and analysis of biological processes and strategies intrinsic to organisms, plants, animals, or ecosystems. Biomimicry unfolds in various branches or approaches, including bioinspired design, engineering, biotechnology, sustainable architecture, and medicine. Biomimicry promotes increased sustainability, innovation, efficiency, adaptability, and resilience while reducing environmental impact. Among the wide range of opportunities and advantages offered by applying this approach is the ease of developing innovative and sustainable solutions that are in harmony with the environment. Consequently, there is a particular interest in integrating this trend into the design, development, and manufacturing of products, i.e., all stages of the design process. The success of applied biomimicry lies first in prior knowledge of the methodologies, tools, and existing knowledge in this field to reduce development times and facilitate the integration of knowledge and decision-making. Secondly, it offers results focused on zero impact, efficiency, and optimization of solutions. Additionally, it serves as a source of inspiration to enhance the creative process and innovation. Figure 1 establishes the correlation between the phases of the design of the product design (needs analysis, functional design, conceptual design, and detail design) and the intrinsic stages of the biomimicry design spiral (define, biologize, discover, and abstract) [4].

By integrating a biomimetic design process and after analyzing the needs, these are transferred to the functional domain following the approach of biologizing potential sustainable solutions guided by biological strategies. This task is carried out using the Biomimicry Taxonomy [5] as a tool that streamlines this process. Once this phase is complete, the conceptual design of the different alternatives begins. This is the most creative stage, with increased potential since nature is an inexhaustible source of inspiration. Key tools include journals, books, or databases such as AskNature [6], which complement the Biomimicry Taxonomy. The exhaustive search for relevant biological knowledge drives innovation in solutions and processes. It is also worth highlighting that inspiration can originate at the organism, behavior, or ecosystem level. Finally, in the detailed design phase, the solution is defined and optimized to assess its suitability and validate the product using various prototypes before commercialization.

It is important to note that the biomimetic approach has been developed and implemented by the scientific and technical community across different fields of knowledge over the years. This approach can be traced back to ancient Greek legends such as that of Daedalus and Icarus, where they imitated the flight of a bird [7]; Leonardo Da Vinci’s Renaissance flying machine [8]; or the invention of Velcro by George de Mestral in the twentieth century [3], among others. Recent studies have shown that using a nature-based approach allows the development of engineering solutions with better results [9]. This has enabled greater integration into scientific research, business and education, leading to significant investment in this rapidly expanding field. One of the most prominent institutions in this area is the Biomimicry Institute [3].

The application of biomimicry has been successfully incorporated into research areas related to technology (ant-based algorithms [10]), medicine (antibacterial adhesive based on shark skin [11]), energy (wind turbines based on insect wings [12]), transportation (route optimization based on bees [13] or drones mimicking natural flyers [14,15,16]), architecture (building climate control buildings based on termites [17]), and industrial design (foldable protective helmet based on turtles [18]), among other examples. In industry, bioinspired solutions can be found in various fields, allowing for the resolution of complex challenges with cutting-edge robotic technology, such as in exploration, shipwreck research, waste cleanup, and rescues in the most unknown and hard-to-reach areas, such as seas and oceans, based on underwater biorobots [19]. A more popular contemporary example is the Airbus A300-600ST or Beluga, a bio-inspired cargo aircraft [20]. Additionally, numerous tools and methodologies have been developed for the application of biomimicry.

Biomimicry is a promising discipline. Its application to technological development is a research hotspot today. Although some fields (such as biomedicine [11], automotive [21], or robotics [22]) have made substantial advances, their application still faces various challenges and limitations. The main drawbacks are related to the biological complexity of nature, making direct replication or translation of its principles into solutions, processes, or structures in industrial applications challenging. Managing this complexity requires a multidisciplinary integration of knowledge (including biology, engineering, and industrial design, among other areas); lack of interdisciplinary skills in the design team can limit effective application. Second, the lack of widely accepted standards and methodologies complicates its consistent application and widespread adoption in design projects. Finally, there are technological limitations related to the industry’s capabilities to replicate biological processes.

In this context, this article explores the progress and research efforts related to biomimicry studies; it analyzes the most applied tools and methodologies in each industrial sector. The main objective is to identify sectors that have experienced greater adoption of biomimicry as well as those where its application is still in its infancy. Furthermore, through different case studies, the advantages and application difficulties of biomimetic methodology and procedures are determined, followed by identifying potential gaps and future research directions necessary for standardization and normalized implementation in the industry. The results provide a comprehensive approach to biomimicry applied to more sustainable practices in product design and technology development. It should be noted that there are studies conducting reviews of the biomimetic literature [23], offering a broad overview of objects and processes of interest found in nature and their applicability [24], evaluating their importance in different industries such as chemical and process industries [25], robotics [26], textile industry [27,28], cultural art [29], construction (advances of biomimicry in structural colors [30] or structure design [31]), and materials science and manufacturing (bioinspired smart materials [32], natural photonic materials [33], structural design elements in biological materials [34], additive manufacturing [35]), as well as reviews on case studies, principles and examples of biomimetic design [36,37,38]. However, updated reviews that provide a structured overview of available methodologies and tools, along with a comprehensive analysis of the main applications of biomimicry in product and technology design, have not been identified.

To achieve this, this work is structured as follows: Section 2 describes the methodology used. Section 3 includes the results of the review, integrating bibliometric analysis, analysis of principles, methodologies, and tools, and the evaluation of the applicability of biomimetic processes in the design of industrial products and technology. Section 4 discusses the main findings, concluding in Section 5 with the conclusions of the study.

## 2. Methodology

**Figure 2:**
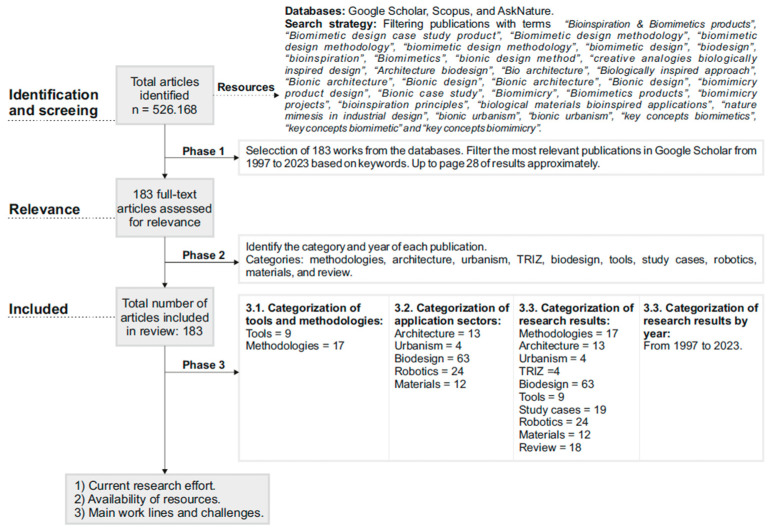
Research methodology, stage 1.

**Figure 3:**
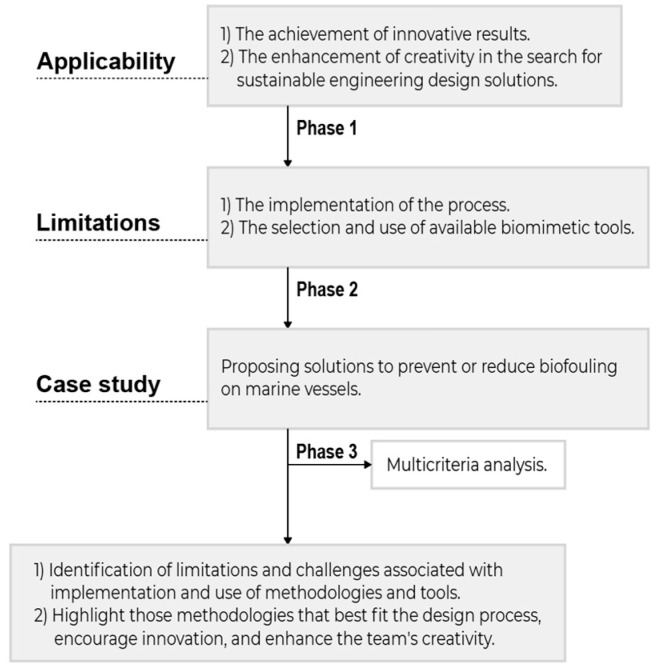
Research methodology, stage 2.

The methodology is divided into two stages (Figure 2 and Figure 3). For stage 1, and in the field of biomimetics, a comprehensive review of the scientific literature has been conducted. The review entails a critical analysis of existing publications, identifying the current state of methodologies, tools, and applications of biomimetics in various domains, as well as future research trends in the field. The review presented in this article is structured around the analysis of three different contexts: (I) bibliometric analysis, (II) a detailed discussion of the advantages of applying biomimetics in industrial product projects, and (III) definitions of future lines of work. The reference database was created from a set of strategically selected search strings. In this study, and with a holistic approach, an initial primary search was conducted in the Scopus, Google Scholar, and AskNature databases [6], dated up to 1997 (the year identified with increasing publication frequency). Data were compiled from a wide range of Web-based sources, including journals, academic articles, books, and proceedings. A total of 183 references were selected for this research using the following keywords: “Bioinspiration and Biomimetics products”, “Biomimetic design case study product”, “Biomimetic design methodology”, “biologically inspired design”, “Biodesign”, “four characteristics biologically inspired design”, “Bioinspiration”, “Biomimetics”, “bionic design method”, “creative analogies biologically inspired design”, “Architecture biodesign”, “Bio architecture”, “Biologically inspired approach”, “Bionic architecture”, “Bionic design”, “Biologically inspired approach”, “Bionic architecture”, “Bionic design”, “natural materials bionic design”, “biomimicry product design”, “Biodesign products”, “Bionic case study”, “Biomimicry”, “Biomimetics products”, “biomimicry projects”, “bioinspiration principles”, “biological materials bioinspired applications”, “nature mimesis in industrial design”, “bionic urbanism”, “key concepts biomimetics”, “key concepts biomimesis” and “key concepts biomimetism”. Additionally, to verify the results obtained in this review, graphical resources of keyword results such as “biomimicry” were consulted, provided by the Scopus bibliographic database. In Phase 1, the analysis and selection of the final sample of relevant publications for this review were carried out. The specific variables considered were: (1) thematic relevance, where publications had to be directly or indirectly related to the design of industrial products and their contribution to sustainable development; (2) application to the industrial context and practical feasibility; (3) publication quality; (4) originality and innovation of proposals.

Stage 2 evaluated the applicability of biomimicry by examining how its principles promote (1) the achievement of innovative results and (2) the enhancement of creativity in the search for sustainable engineering design solutions. Additionally, the current limitations posed by (1) the implementation of the process and (2) the selection and use of available biomimetic tools were identified. This phase was carried out using the procedure indicated in Figure 3; the main methodologies identified in Stage 1 were applied to a case study: propose solutions to prevent or reduce biofouling in marine vessels. The results were used to evaluate, comparatively, the applicability of the methodologies through a multi-criteria analysis. This analysis allowed identification of the limitations and challenges associated with implementation and use, as well as highlighting those methodologies that best fit the design process, foster innovation, and enhance the team’s creativity.

## 3. Results

### 3.1. Bibliometric Analysis

**Figure 4:**
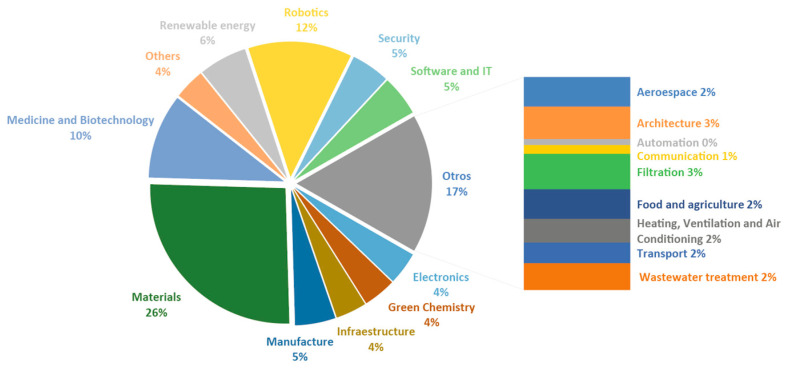
Application sectors according to AskNature.

Firstly, a general analysis on innovative developments in biomimicry was conducted using data published on the specialized biomimicry platform known as AskNature [6]. Specifically, the values corresponding to the innovations filtered by sector are of interest, as shown in Figure 4. It should be noted that most of the results relate to innovations in the field of materials engineering, with 113 publications accounting for 26% of the total data. Following this are categories such as ‘Robotics’ and ‘Medicine and biotechnology’, comprising 12% and 10%, respectively. However, there are areas where, up to now, very few nature-inspired innovations have been addressed, such as automation or communication. Among the directly influential factors contributing to the success of these results are the need for environmentally sustainable materials and resources, as well as the increasing technological development in recent years. Additionally, medicine and biotechnology are closely related to nature and learn from it to solve or remedy real problems. Research on new biomimetic materials allows for easy applicability across various sectors and is socially well-received, contributing to increased project value regardless of the application area.

**Figure 5:**
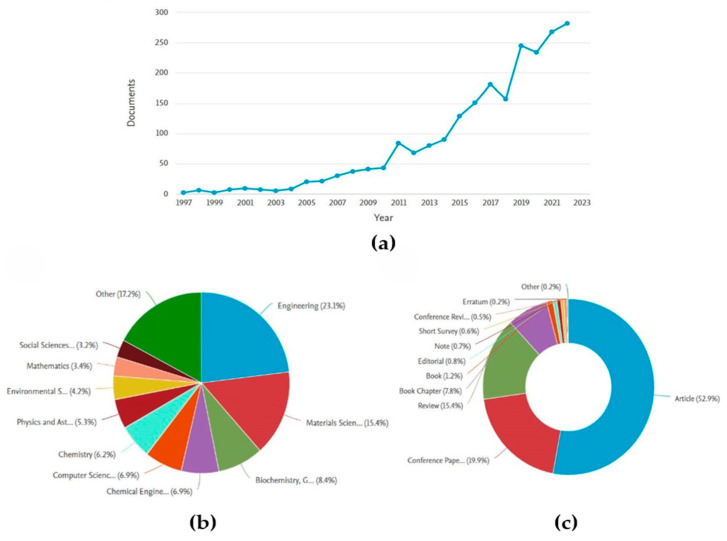
(**a**) Biomimicry publications by year of publication; (**b**) Results categorized by sector; (**c**) Results according to the type of publications.

Second, scientific publications were analyzed; as mentioned in Section 2 of the methods, the search was performed through the Scopus bibliographic database [39], which allows graphical display of the search results. In this case, it was decided to filter the published results related to the term “biomimicry” from 1997 to the present. As shown in Figure 5a, there is a growing trend in biomimicry research and development. Furthermore, the classification shown in Figure 5b was performed, where the results were categorized by sector (left) and type of publication (right). From these, it can be deduced that the field with the greatest applicability is engineering, that most related publications are natural scientific articles, and that there is a justified growing trend in biomimicry research.

Finally, the selected sample of 138 publications was analyzed, emphasizing the research efforts undertaken by the scientific community in recent years. Firstly, the scope of the work developed is underscored, focusing on (i) proposals of biomimetic methodologies, frameworks, and principles; (ii) tools that aid in the implementation of the methodology; and (iii) case studies. As depicted in Figure 5 (left), there is evident interest in methodological development.

**Figure 6:**
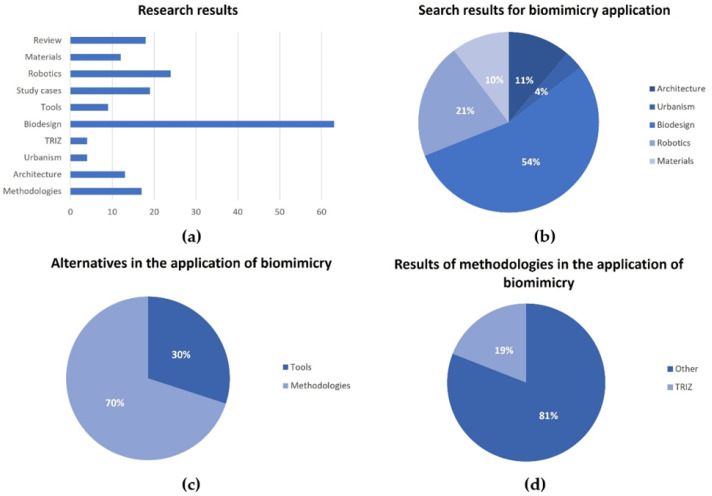
(**a**) The scope of the investigation; (**b**) Applications of biomimicry according to area of knowledge; (**c**) Proposals for methods and tools; (**d**) Selection of methodologies.

Furthermore, the publications were classified into the following areas, which were easily identified by analyzing similarities in the scope of the research and creating different clusters: (1) architecture, (2) urbanism, (3) biodesign, (4) robotics and automated technologies, and (5) materials engineering. Figure 6 shows the results according to this classification. These findings reflect a growing interest in the field of “biodesign” accounting for a categorical percentage of 54%, followed by other application areas such as robotics, architecture, materials, and urbanism, which represent values of 21%, 11%, 10%, and 4%, respectively. Furthermore, in Figure 6 on the right, the complete set of results is depicted in a bar graph according to their nature, where the prominence of applications based on “biodesign” is once again highlighted.

**Figure 7:**
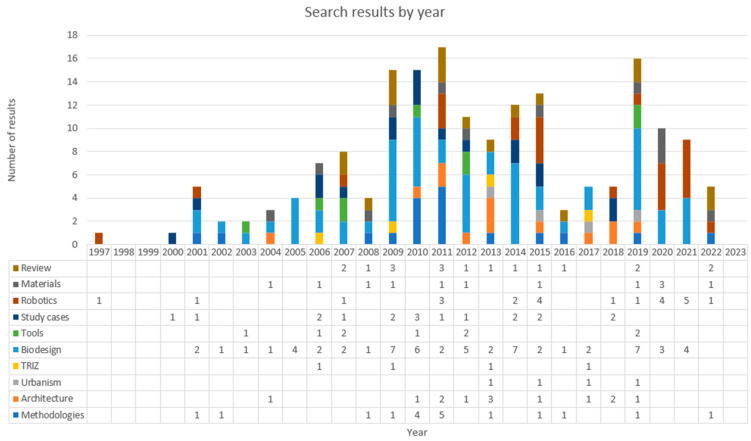
Results classified by area of knowledge and year.

Finally, Figure 7 presents the results compiled by year. It describes a certain trend toward publications related to the field of biodesign in the early 21st century. It is not until the middle of the first decade that there is more significant knowledge about tools and methodologies framed in this subject. From the second decade onward, more consolidated publications on architecture, urbanism, and robotics begin to appear. In particular, there is a significant presence of content related to case studies over time, indicating ongoing research interest in this topic and prompting an increase in the number of reviews conducted for each category.

The results of the bibliometric study reflect, in general terms, that approximately 70% of the analyzed publications focus on the application of biomimicry in specific projects, products, and systems, while the remaining 30% address the development of methodologies and tools to facilitate the implementation of biomimicry in the design and development process (see Figure 6c). It should be noted that among the wide variety of methodologies used, TRIZ (Theory of Inventive Problem Solving) is the most frequently applied (see Figure 6d).

Furthermore, the classification of publications (see Figure 6b) shows a significant distribution in the application of biomimicry in various fields of knowledge. Biodesign leads with 54%, being a universally used strategy for creating sustainable solutions inspired by nature. Architecture represents 15% of the applications, within which biomimetic urbanism is highly relevant (4%). Similarly, robotics has also seen significant adoption at 21%, followed by material design at 10%. This diversity of fields of knowledge and the results of each scientific publication highlight the versatility of biomimetic methodologies and their potential for innovation in a variety of scientific and technological fields.

Table 1 presents a summary of the bibliometric study, the keywords used for the search, and the most representative bibliographic references.

### 3.2. Biomimetics: Analysis of the Fundamentals and Available Framework

This section analyzes the current state and scope of the fundamental concepts of biomimetics, the essential principles guiding this discipline, and the framework for its application.

The analysis of the results revealed that publications frequently use the terms biomimetics and biomimicry interchangeably. Although both terms refer to the use of biology as a source of learning and a reference to mimic and develop design solutions, there are nuances that distinguish them in terms of their focus and methodology [164]. Biomimetics focusses on mimicking biology to produce creative solutions based on the analogy of biological phenomena (for example, designing a bullet train inspired by the streamlined shape of a kingfisher’s beak; geometry that reduces wind resistance, noise, and improves train speed [189]); whereas “biomimicry” focuses on applying biological knowledge to develop sustainable practices (for example, developing a water purification system inspired by the filtration ability of fish gills [190]). Table 2 lists the main terminology classified into categories and subcategories for the two approaches.

Biomimicry takes nature as a source of learning and inspiration, grounding its processes in three fundamental principles: emulating (creating regenerative designs through the study and replication of nature), ethics (understanding the workings of life to create designs that are not harmful), and (re)connecting (rebuilding the harmonious and respectful relationship between humans and nature) [191]. Biomimicry also involves partial or complete imitation of forms, materials, structures, processes, or functions found in nature, considering three levels of application: organism, by imitating the specific physical and biological characteristics of living beings; behavior, translating how organisms act and interact with their environment; or ecosystem, studying their functioning as self-sustaining entities (adaptive, self-organized, self-regenerative, and self-optimized) [51].

It should be noted that human needs have been met by the set of phenomena, ecosystems, or living beings that exist in nature without altering natural cycles and contributing to the construction of a planet that has been integrated and functioning for millions of years. However, the interpretation of nature patterns and their application to viable engineering solutions is complex.

To address this challenge, a variety of research endeavors aim to standardize the creation of analogies between natural processes and human processes. Benyus [192] developed a framework to explore innovative and sustainable solutions using nature. McDonough and Braungart [193] introduced Cradle to Cradle (C2C), a paradigm focused on architecture and product design. Industrial ecology [194], on the other hand, aims to develop industrial systems that mimic natural systems, identifying interaction patterns, exchange flows, and the properties that industrial systems must exhibit as ecosystems. Finally, Riechmann’s research [195] deserves mention, as it explores the principle of biomimicry from a broad perspective, allowing for an understanding of the operational principles of life at various levels. The ecosystemic perspective highlighted there contributes to the reconstruction of human ecosystems to be fully integrated with natural ones.

In general, all of these biomimetic frameworks start from the strategy of using nature as a reference, considering it (1) as a mentor, that is, a source of knowledge and experience in efficient and effective principles and phenomena that can guide the design of sustainable systems; (2) as a model from which requirements and solutions to imitate are extracted, such as forms, processes, systems, and strategies, through a process of transposition; and (3) as a measure, representing the domain of analysis of solutions, as a space for comparison between natural and artificial models, facilitating the evaluation of technological innovations through the application of ecological standards [192,193]. Table 3 compares the most relevant approaches currently existing, adapted from the common framework or from generic phases.

**Figure 8:**
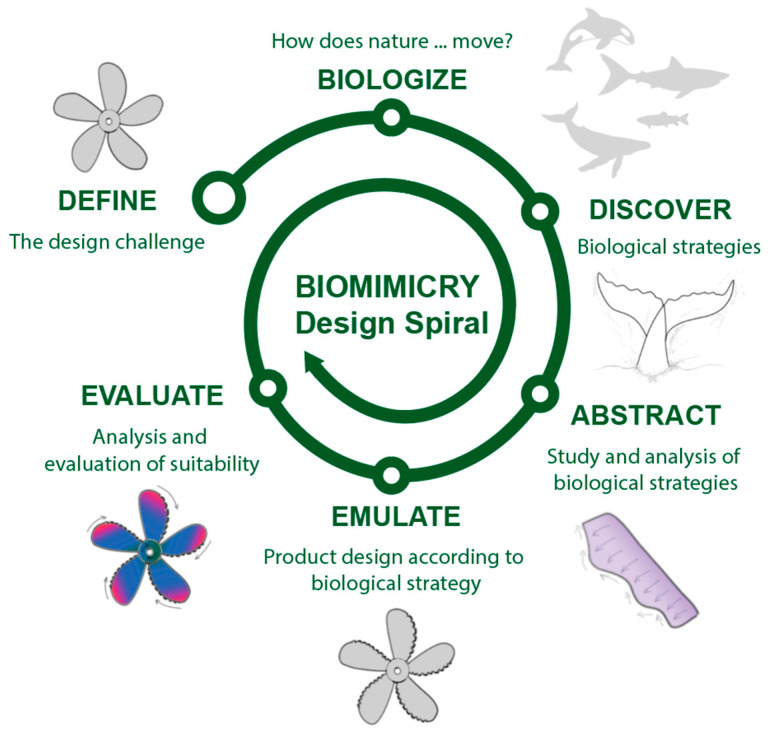
Biomimicry Design Spiral.

The general stages of the process are illustrated in the design spiral of Figure 8 (adapted from the methodological approaches compared in Table 3). One of the fundamental and most complex stages is defining the specific function required and identifying the context in which the design will be applied. This involves biologists, that is, considering how nature performs the desired function and then identifying the most suitable biological models. There are databases of biological strategies that allow for the exploration of solutions based on the challenge to be addressed [6]. During the abstraction phase, the characteristics or mechanisms of the selected biological strategies are studied in detail; the use of sketches is a useful tool in this step, facilitating the understanding and visualization of these strategies. Emulation requires an analysis of patterns and relationships of identified biological strategies, where the creation of conceptual and mental maps, and rapid prototyping, can provide clarity and organization to the process. Finally, the evaluation phase focuses on the critical review of the proposed design, considering its social, environmental, technical, and economic viability [6]. There are various useful evaluation tools in this phase, such as Life Cycle Assessment (LCA), selection matrices, or computational simulators. The general stages of the process are illustrated in the design spiral shown in Figure 8 (adapted from the methodological approaches compared in Table 3). One of the fundamental and most complex stages is defining the specific required function and identifying the context in which the design will be applied. This involves biologizing, meaning considering how nature performs the desired function in order to identify the most suitable biological models.

It should be noted that the process of applying biomimicry requires a high degree of knowledge of biological systems. Specialized methodologies and tools are created to streamline and optimize this process. They are selected based on project requirements and specifications, facilitating the translation of biological knowledge into technological applications. These methodologies (such as BioTRIZ [196], MBE [197], UNO-BID [198], DANE [199], SAPPhIRE [200], or Bio-SBF [201]) allow for addressing specific application problems through sequential and systematic processes that improve the applicability of biomimicry in design and engineering.

Two main groups of methodologies are distinguished: textual and schematic. Textual methodologies are based on the description of biological knowledge using nouns, verbs, and prepositions. They require an important level of understanding of biological principles but are not adequately adapted to the process of technological design and development. Within this type of methodology are AskNature and Biomimicry 3.8; also included in this group are all bibliographic sources such as books and scientific publications that describe biological principles and case studies applying them.

However, schematic methodologies are based on graphical representation to illustrate the structures, functions, behaviors, and interrelationships within biological systems. Although they do not require prior knowledge of biology, they require skills in modeling, analysis, and design of functions. This group includes DANE (Design by Analogy to Nature Engine), Bio-SBF (Bio-System Based Framework), SAPPhIRE, UNO-BID (Universal Nominal Biologically Inspired Design), and MBE (Model-Based Engineering); their graphical approach facilitates the translation from biological concepts to technological solutions. DANE uses the traditional method to express biomimetic functions. SAPPhIRE is based on identifying causal relationships between elements, whereas UNO-BID employs dynamic physical parameters to identify relevant biological properties. Finally, MBE establishes terminology based on general attributes to abstract innovative knowledge and apply it to prototypes.

Within the spectrum of available resources, some such as BioTRIZ, AskNature, Design Spiral, DANE 2.0, or idea-Inspire are based on a hybrid approach that includes textual strategies and schematic representation to facilitate the application of biomimetic design. The Design Spiral stands out for its intuitive approach to conceptualizing solutions in projects, stimulating creative thinking in the initial stages. However, by using BioTRIZ in combination with other design tools and databases such as AskNature [6], results based on quantitative and practical parameters can be achieved, simplifying the abstraction from theory to technical realization. Similarly, MBE (Model Based Engineering) [164], linked to TRIZ, offers a more structured approach to biomimetic design. Although this methodology was not specifically developed for biomimetic contexts, it can be used to guide the development of solutions by facilitating problem analysis and definition, translating it into biological terms, classifying and comparing different bioprototypes, analyzing biological strategies and their technological application, and finally, implementation and verification. It should be noted that while DANE 2.0 and Idea-Inspire are mentioned in numerous publications as tools applicable to biomimicry, the difficulty of accessing these platforms limits their utility. Table 4 analyzes the applicability of these methodologies according to their suitability for each stage of the biomimicry framework.

The application of these methodologies can present a certain level of abstraction that makes their application challenging. Therefore, they often rely on tools that streamline the procedures, including the development and application of software, modeling and simulation, evaluation and checklists, databases, and selected materials, among other innovative tools aimed at improving design and engineering through biomimicry. The research and proposals of The Biomimicry Institute [3] stand out in this group. The biomimicry taxonomy offers a classification of biological strategies based on the required function. This resource is complemented by access to AskNature [6]. Although these resources are highly functional, it is important to consider the specific context in which the solution will be applied, as different contexts may require different strategies to achieve the same functionality. Table 5 summarizes the biomimicry taxonomy, listing specific functions, along with application examples. The complete study can be found in the Supplementary Materials.

**Figure 9:**
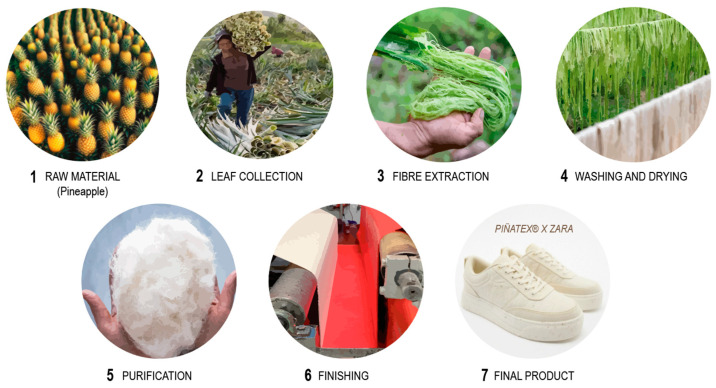
The Manufacturing Process of Piñatex.

However, there are a wide variety of tools that facilitate the application of biomimicry in the design process. These include databases, material selectors, consulting companies or organizations, scientific journals, and other sources. Table 6 shows some of the most relevant tools. These provide knowledge and inspiration on biomimetic applications, such as the collaboration between Zara and Piñatex^®^ (a material made from pineapple leaf fiber) [202]. Figure 9 shows the manufacturing process of this sustainable material, along with one of the commercialized results of this collaboration [203].

### 3.3. Evaluation of the Applicability of the Biomimetic Procedure

This section evaluates the applicability of biomimicry. It analyzes how its principles promote (1) the achievement of innovative results and (2) the enhancement of creativity in the search for sustainable engineering design solutions. In addition, it identifies the current limitations posed by (1) the implementation of the process and (2) the selection and use of available biomimetic tools. The selected case study was the search for solutions to control (prevent or reduce) biofouling in marine vessels.

The technological systems operating in oceanic and maritime environments for research and exploration maintain direct contact with the marine ecosystem, experiencing the natural phenomenon of biofouling [216]. Various organisms, such as algae, barnacles, and mollusks, adhere to submerged surfaces, affecting the durability of structures, negatively impacting aesthetics, and increasing resistance to vessel advancement (resulting in increased fuel consumption related to greenhouse gas emissions). Additionally, in the past decade, the introduction of robotic systems has been promoted to streamline operations, reduce operational and labor costs, and improve occupational risk control. Biofouling is a serious problem for these unmanned vessels, as it can obstruct mechanical components and electronic systems, compromising their operation. Furthermore, the design of these systems [217,218,219] can involve environmental risks such as habitat alteration, oil, fuel, or chemical waste emissions pollution, noise pollution, disruption of migration or feeding patterns, thermal effects, and modification of ocean currents. For these reasons, the use of biomimicry in the design of these systems has allowed for a reduction in environmental impact, optimizing aspects such as propulsion, stability, and maneuverability, among others. This clear interest in the search for technological solutions for the marine environment based on nature has led to the evolution of these systems into biologically inspired autonomous underwater vehicles (BAUV).

**Figure 10:**
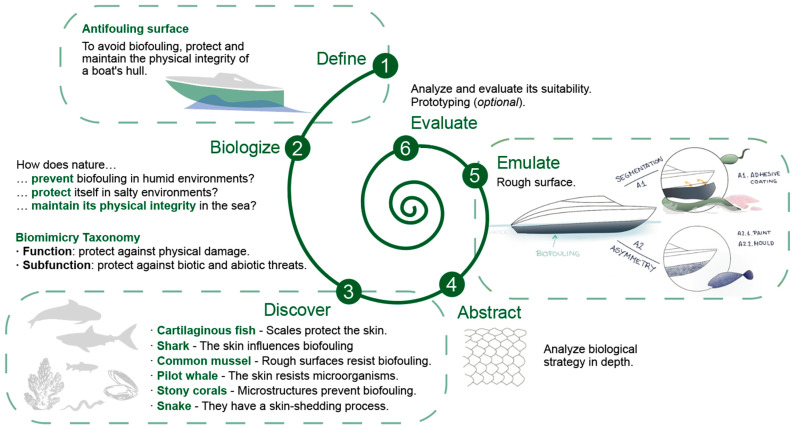
Design Spiral results.

For the development of bioinspired solutions, the Biomimetic Design Spiral [220], BioTRIZ [196] (due to its analogy to the TRIZ methodology, widely known in engineering for creative problem solving), Biomimicry Taxonomy [5] and AskNature [6] were selected. The latter facilitated the identification of the most relevant biological strategies according to the specific function to be addressed (biofouling) in the context of use (marine environment). Other methodologies or tools, such as DANE 2.0 and Idea-Inspire, were discarded (due to their level of update, accessibility, or compatibility), as well as SAPPhIRE (due to the high degree of definition of this causality model, which would be interesting if access to the Idea-Inspire tool were available), and Bio-SBF and MBE (due to requiring a higher degree of definition). UNO-BID was also excluded (being a combination of DANE and SAPPhIRE, requiring greater definition and understanding), along with the free and open access ontology software Protégé v.5.6.4 [221], to achieve an optimal result. Analyzing the objective of technological development (biofouling control in vessels) in the context of use (marine environment), the biomimetic design spiral (define, discover, abstract, emulate, and evaluate) was applied, effectively guiding the stages and simplifying the necessary iteration between the phases of observation, abstraction, emulation, and evaluation. Figure 10 summarizes the results of the spiral.

The problem to be solved is posed by the following question: How can we prevent biofouling on a vessel? The context must be properly defined: the marine environment. The answer begins with the biological phase (Table 7), where questions are formulated about how nature performs similar functions.

In the discovery phase (or search for biological strategies), the AskNature resource [6] was used to explore how nature addresses the issue of biofouling. This platform facilitated the identification of organisms that have evolved with surfaces that repel the adhesion of other living beings or exhibit behaviors resistant to this phenomenon. Subsequently, in the abstraction phase, the biomimetic taxonomy [5] was used to decode the underlying principles of natural strategies and translate them into technologically applicable design solutions. Taxonomy streamlines the search in the AskNature database [6] and incorporates Biomole [222], an add-on that identifies coexisting functions and streamlines research. In this case, the function “protect against physical damage” and subfunctions related to protection against biotic and abiotic threats were selected. Table 8 provides a synthesis of the biological strategies extracted from the database.

**Figure 11:**
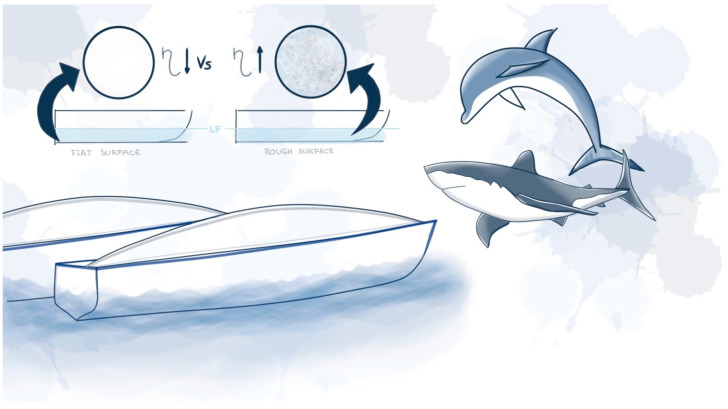
Alternative generated with the biomimicry taxonomy.

Figure 11 illustrates the brainstorming for solutions involving paints that mimic shark skin or scales. Before moving on to the prototyping phase, it is important to perform a systematic evaluation of the proposal. For this purpose, the Biomimicry Institute offers a useful checklist [223].

Applying this checklist emphasizes that the designed solution should be manufactured locally, using recyclable or recycled materials that allow reconfiguration. It should exhibit multifunctional characteristics for application in various areas, redefine a competitive advantage based on nature-inspired strategies, and avoid toxic and polluting materials. These are some aspects that easily fulfill the proposed solution, but others included in this checklist related to product marketing or the competent company or organization have not been considered because validation has been limited to the product-solution design.

On the one hand, the BioTRIZ method was applied, which is an adaptation of the TRIZ methodology for inventive problem solving [196]. BioTRIZ relies on 40 inventive principles (Table 9) and various associated parameters (Table 10), arranged in a BioTRIZ contradiction matrix (Table 11), to identify and overcome obstacles that may arise in the design process. Although it is possible to apply the QFDE (Quality Function Deployment) matrix beforehand to translate needs into technical specifications, this was not considered because of the study’s objective (evaluating the applicability of biomimetic methodologies). Once the problem was identified, the area to be improved and the obstacle factor were selected; in the case study, these were structure and time, respectively. Based on this selection, the most relevant inventive principles are 1 (segmentation), 2 (extraction), and 4 (asymmetry), as detailed in Table 11.

**Figure 12:**
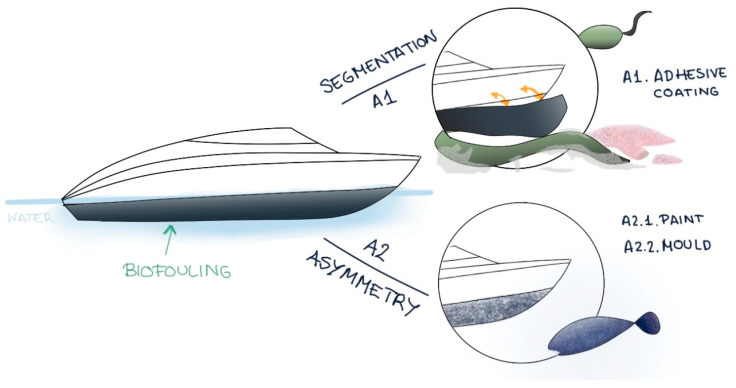
Alternatives generated with BioTRIZ.

In this context, the highlighted strategies include dividing the hull into independent and easily removable parts, both superficially and structurally, as shown in Figure 12. To generate biomimetic alternatives aligned with these principles, it was necessary to consult various sources, such as specialized journals, books, and biomimetic databases, to gather information on relevant biological strategies [224,225,226,227,228,229].

From this analysis, possible inspirations for segmentation are drawn, based on the ability of snakes to shed their skin [229], or the ability of starfish to detach and regenerate their limbs when attacked, even generating a new body structure from a detached limb [164]. The first alternative is proposed, involving the application of an easily removable layer on the lower part of the vessel’s hull, below the waterline. This innovative approach would not only facilitate biofouling removal but could also be reused for other purposes. The principle of asymmetry can be applied by introducing surface roughness into submerged areas, creating molds or paints that mimic fish scales. An example of this solution is antifouling paints based on shark skin [226].

Finally, after generating alternatives, it is necessary to evaluate the feasibility and suitability of the solutions. For this purpose, it is necessary to resort to other complementary methods, as the validation stage is not included in BioTRIZ [58,196]. Specialized biomimicry validation methods can be used (such as the Biomimicry Institute Checklist [223], which provides a specialized procedure to ensure solutions are aligned with ecological and sustainability principles) or alternative non-specialized evaluation methodologies (such as Analytic Hierarchy Process (AHP) or multi-criteria analysis, which assess feasibility from multiple perspectives including technical, economic and environmental factors). Additionally, a Life Cycle Assessment (LCA) can be useful for verifying and comparing the environmental impact of solutions.

#### Evaluation of Biomimetic Methodologies

To assess the applicability of the methodologies, a multi-criteria analysis was conducted (Table 12), which allowed for identifying the limitations and challenges associated with implementation and use, as well as highlighting those that best fit the design process, fostering innovation and improving the creativity of the team. The methodologies were evaluated using a set of semiquantitative indicators:

- Accessibility of tools: ease of search; open access/private license; ease of download and installation.
- Implementation time: duration of the design process from conceptualization to prototyping.
- Technical barrier: technical obstacles encountered during implementation, including limitations in the database, data availability and accessibility, materialization capability (prototyping, simulation).
- Theory-practice gap: difference between expected results based on biomimetic theory and actual results obtained in practical applications.
- Integration with conventional design and development processes: integration compatibility.
- Usability according to resource nature: availability of interactive and digital platforms; or static resources (such as manuals, PDF guides, or checklists).
- Required knowledge level, adaptability to users: need for specialized knowledge in biological systems; intuitive application by professionals without specialization in underlying biological principles.

Among the methodologies and tools applied, the Design Spiral [223] stands out as a valuable initiation into biomimicry, fostering creative thinking under this approach. However, combining BioTRIZ [58,196] with other design tools and search engines such as AskNature [6] can produce a more comprehensive and defined outcome. BioTRIZ excels in the emulation phase because of its defined design principles, facilitating the translation of these principles into specific technical solutions. DANE 2.0 and Idea-Inspire are mentioned in numerous publications as tools for biomimicry applicability [230,231,232], but currently face accessibility problems.

## 4. Discussion

The results of the literature review show a growing trend toward the adoption and development of knowledge in engineering, materials science, and architecture, highlighting substantial progressive advancements in biodesign, especially in robotics. The interest in biomimicry as a tool for technological advancement in engineering is driven by the need to enhance the functionality, efficiency, and sustainability of the proposed solutions. The areas that experienced the greatest growth included robotics, primarily for exploration, rescue missions, and agriculture tasks; and the energy sector, focused on optimizing the development and efficiency of renewable technologies. In addition, the development of bioinspired materials and manufacturing methods has significant applications in the medicine, construction, and aerospace industries. In architecture, biomimicry emerges as a tool for creating more sustainable environments and improving adaptability, durability, and aesthetics.

This trend is driven, first, by the advantages that biomimicry offers at a strategic level for the organization; significant improvements are identified in planning processes and project success achievement. Particularly notable is the enhancement in the scope and optimization of timelines, allowing for accelerated development times. Biomimicry acts as a catalyst for creativity, fostering the generation of innovative ideas and opening new business opportunities. In this way, the organization is on a strategic path of sustainable innovation. At the operational level, compared to traditional design methods, biomimicry significantly enhances the social and environmental performance of developed technological solutions, including its adaptability and resilience to environmental conditions, energy efficiency, and a significant reduction in environmental impact.

In contrast, there are sectors where biomimicry has not achieved widespread applicability, such as automation, wastewater treatment, HVAC and communications, with few developments. This situation can be attributed, in part, to the challenges associated with research and a deep understanding of biological principles, as well as the complexity of abstracting them into viable and scalable solutions. Therefore, the main barrier identified in this research is the need for multidisciplinary teams that combine biological knowledge with specific technical competencies in each sector. Currently, it is not common to find industry teams that integrate specialized profiles in natural environments and ecosystems; this poses an obstacle to the adoption of biomimetic approaches.

Despite these difficulties, biomimicry can be an opportunity to achieve sustainable development goals in the industry. It should be noted that progress in the SDGs generally faces obstacles mainly due to the lack of alignment between traditional engineering methods and emerging sustainable approaches. This discrepancy arises from the challenge of balancing economic feasibility with desired outcomes in terms of environmental and social impact. Adopting integrated solutions or proposals that aim to simultaneously improve social impact, environmental performance, and quality, along with technical and economic feasibility, represents a challenge for companies and organizations. These strategies often involve high complexity in management, substantial initial investments, and subsequent maintenance costs. At times, sustainable proposals can compromise established economic and technical objectives. Similarly, after implementation, clean technologies demand new and specific specialized operational knowledge. Additionally, it must be noted that often the benefits materialize in the medium to long term and not always in monetary gains. Biomimicry can help overcome these obstacles by streamlining and providing flexibility in the design and manufacturing process of sustainable solutions, balancing social, environmental, and economic dimensions. To achieve this, it is necessary to establish a framework complemented by agile tools and comprehensive databases. However, none of the biomimicry approaches and methodologies available allow for a comprehensive integration of all phases of an engineering project from planning and design to modeling, simulation, optimization, or analysis. Similarly, while various resources are available, these are independent of each other, which complicates their combined application within the same project. Therefore, it is essential to unify and simplify the procedure by gathering user guides, methodology, tools, and digital resources that are easily accessible. It also underscores the current challenge of seeking and accessing digital resources, as well as selecting the most suitable ones based on the objective and expected outcome.

The analysis carried out in this research reveals several strategic directions for biomimicry research, which will maximize its value and applicability in the industrial sector.

- Development and establishment of a standardized framework: create a standard framework to guide the biomimetic process coherently, intuitively, and easily integrable with other procedures for analyzing social, economic, and environmental impacts (such as Life Cycle Assessment).
- Development of an integrated procedure that combines a variety of complementary resources, including methodology, technical guidelines, and support tools such as databases and evaluators. The procedure should be easily integrated into conventional technological development approaches.
- Enhancing the availability and access to quality biomimetic resources, including expanding and updating databases, specialized software, or compilations of case studies that can serve as references.
- Finally, beyond the research sphere, it is important for organizations to promote the formation of multidisciplinary teams with experts in biological principles. Additionally, creating these teams is more effective from the initial stages of engineering education at universities, where competencies related to the proper mimetic integration of technology into ecosystems are included.

## 5. Conclusions

Nature has provided answers to a variety of phenomena, processes, and structures with minimal complexity, optimizing outcomes over millions of years of evolution. This fact underscores the interest in using nature as a reference to develop industrial ecosystems similar to natural ones, addressing the central problem of our time: the clash between industrial development and the biophysical limits of the planet. In this context, biomimicry emerges as a versatile strategy that can be applied in all engineering fields. Despite the significant body of knowledge currently available, seeking and using resources often presents challenges. There is a growing interest in creating unified platforms that facilitate access to methodologies, databases, evaluation tools, and other digital resources to simplify the application of biomimicry in engineering projects. The goal is to enhance and accelerate the proposal and implementation of more sustainable and innovative solutions.

## Figures and Tables

**Table 1:** Summary of bibliographic sources.

Category	Keywords	Reference
**(1) Classification according to areas of knowledge**		
Architecture	Architecture; Architecture biodesign; Bionic architecture; Biophilic architecture; Biomimetic materials in architecture; Sustainable architecture; Biomimetic building design; Ecological, Biomorphic, Organic architecture.	[40,41,42,43,44,45,46,47,48,49,50,51,52]
Town planning	Bio urbanism; ecological urban planning; nature-based urban design; sustainable urban development; biophilic cities; resilient urban infrastructure; green infrastructure planning; ecosystem-based urban design; urban biodiversity conservation; regenerative urban design.	[53,54,55,56]
TRIZ	Inventive problem solving; systematic innovation; inventive principles; four characteristics of biologically inspired design; Bioinspiration & Biomimetics products; Biomimetic design case study.	[36,57,58,59]
Biodesign	Creative analogies; biologically inspired design; biodesign products; nature-inspired design; bioinspiration; biomimetics products.	[24,25,29,60,61,62,63,64,65,66,67,68,69,70,71,72,73,74,75,76,77,78,79,80,81,82,83,84,85,86,87,88,89,90,91,92,93,94,95,96,97,98,99,100,101,102,103,104,105,106,107,108,109,110,111,112,113,114,115,116,117]
Robotics	Biomimetic robotics; nature-inspired robotics; nature-inspired mechanisms; robotic systems inspired by animals; biomimetic locomotion.	[22,26,37,118,119,120,121,122,123,124,125,126,127,128,129,130,131,132,133,134,135,136,137]
Materials	Biomimetic composites; bioinspired coatings; nature-inspired polymers; biomimetic structural materials; bionic ceramics; biologically inspired textiles; natural material replication; bioinspired surface modifications; biofabricated materials.	[31,32,33,138,139,140,141,142,143,144,145,146]
	**(2) Classification according to study scope**	
Review	Analysis of the current state of research, identification of trends, synthesis and comparison of methodologies, and future recommendations for biomimetic research.	[23,24,25,26,27,28,29,30,31,32,33,35,36,37,38,147]
Methodology	Proposition of new methodologies and frameworks to address specific application problems; these methodologies enhance the applicability of biomimicry in design and engineering.	[148,149,150,151,152,153,154,155,156,157,158,159,160,161,162,163,164]
Tools	Development and application of software, modeling and simulation, evaluation and checklists, databases, selected materials, among other innovative tools aimed at improving design and engineering through biomimicry.	[34,165,166,167,168,169,170,171]
Case study	The practical application of biomimicry in various contexts; they provide concrete examples where biomimicry has been used to solve specific design and engineering problems.	[172,173,174,175,176,177,178,179,180,181,182,183,184,185,186,187,188]

**Table 2:** Hierarchies of terminology: biomimicry and biomimetics.

Concept	Category	Subcategory
Biomimicry	Organism Behavior Ecosystem	Patterns
		Materials
		Structures
		Processes
		Functions
Biomimetics	Of the construction	Material
		Substance
		Prosthodontics
		Robotics
	Of processes	Energy
		Architecture
		Sensors
		Kinematics
	Of the information	Neuronal
		Evolutionary
		Process
		Organizational

**Table 3:** Comparison of six methodological approaches to biomimetics.

Generic Phases	Gramman (2004) [152]	Schild et al. (2004) [152]	Hill (1997, 2005) [152]	Helms et al. (2009) [152]	Nagel et al. (2011) [153]	Chen et al. (2017) [57]
DEFINE AND BIOLOGIZE (1) Formulate a goal search problem.	(1) Formulate a search objective.	(1) Problem formulation that includes success factors, contradictions, and customer views.	(1) Analyze conflicting demands to determine basic functions.	(1) Problem definition: identify functions, subfunctions, and optimization problems.	(1.1.) Definition of the problem. (1.2) Decompose the needs.	(1) Identification of keywords related to the biology of the product design of the BOP pyramid.
DISCOVER (2) Search for biological analogues.	(2) Search and map a set of relevant biological systems.	(2.1) Evaluate: Is the search for analogies promising? (2.2) Search for analogies in social networks or databases.	(2) Identify relevant biological structures.	(2) Search for biological solutions.	(2) Search for functional biological solutions.	(2) Biological case search and resource analysis.
ABSTRACT (3) Analyze the biological system.	(3.1) Analyze the biological system. (3.2) Evaluate the system to determine if a transfer is possible; if not, review the previous steps.	(3) Verification: is the analog system well understood?	(3) Analyze biological structures: extract basic principles, associate preliminary solutions.	(3) Define the biological solution.	(3) Make connections between biology and engineering.	(3) Choosing the appropriate biological case.
EMULATE (4) Transfer.	(4) Implement an analogy.	(4) Assess transferability: Four levels of transfer are proposed.	(4.1) Transfer preliminary solutions to technical solutions. (4.2.) Vary and combine the relevant characteristics of these solutions.	(4) Application of the principle.	(4.1) Conceptual design of solutions. (4.2) Development of alternatives.	(4) Transfer
EVALUATE (5) Evaluation, verification.	-	-	(5.2) Use common evaluation methods. (5.3) Evaluate the solution chosen.	-	(5) Validation	(5) Evaluation.

**Table 4:** Methods available to solve biologically inspired problems.

Stages	BioTRIZ	MBE	BID	DANE	SAPPhIRE	Bio-SBF
Problem analysis	X	X	X	X	X	X
Define problems abstractly		X	X	X		
Transport to biology	X	X	X		X	X
Classify possible bioprototypes	X	X		X	X	X
Compare and select bioprototypes		X		X	X	X
Analyze biological strategies	X	X	X	X	X	X
Transport to technology	X	X	X			X
Implement and verify		X				

**Table 5:** Biomimicry taxonomy.

Group	Subgroup	Functions	Example
Move or stay put	Attach	Permanently, temporarily.	[120,124]
	Move	In/on solids, in/on liquids, and in/through gases.	[96,118,120,126,127,129]
Protect from physical harm	Protect from living threats	Animals, plants, fungi, and microbes.	[132,140,141]
	Protect against nonliving threats	Excess liquids, loss of liquids, loss of gases, light, temperature, wind, gases, dirt/solids, chemicals, fire, ice, and nuclear radiation.	[31,41,43,140,141]
	Manage structural forces.	Shear, compression, thermal shock, impact, tension, turbulence, mechanical wear, chemical wear, and creep.	[31,41,43,44,140,141,174]
	Regulate physiological processes	Cellular processes, maintenance of homeostasis, and reproduction or growth.	[25]
	Prevent structural failure	Buckling, deformation, fatigue, melting, and fracture/rupture.	[41,138,140,141,174]
	Coordinate	Coordinate by self-organization, activities, and systems.	[55,132]
Maintain community	Cooperate	Interactions within and between species, ecosystems, and systems, including cooperation and competition.	[26,55]
	Provide ecosystem services	Managing disturbances, regulating flows, pollination, soil generation, detoxification, erosion control, nutrient cycling, climate regulation, seed dispersal, biodiversity maintenance, and biological control.	[109,179]
Modify	Modify the physical, chemical, and electrical state	Involves alterations in size, shape, mass, volume, pressure, density, phase, buoyancy, and other material characteristics and adjustments in energy, reactivity, concentration, electrical charge, and other chemical properties.	[31,139]
	Adapt/optimize	Genotype, phenotype, co-evolve, and behaviors.	[31,32,41,55,131,182]
	Transform/convert energy	Conversion of electrical, magnetic, chemical, mechanical, thermal, and radiant energy.	[32]
Make	Reproduce, physically and chemically assemble	The ability to self-replicate; construction of physical and chemical structures, including polymers, metal-based compounds, molecular devices, crystals, inorganic and organic compounds, and modification of chemical bonds on demand.	[41,140,144]
Process information	Navigate	Movement through air, liquid, solid, and land.	[132]
	Sending signals	Various means such as light, sound, touch, and chemicals.	[32]
	Processing signals and compute	Includes differentiating, transducing, and responding to signals. Computing, learning, and decoding.	[31,37,119,123]
	Sensing environmental cues	Numerous factors such as light, temperature, motion, and time.	[25,31,32,96]
Break down	Chemically and physically break down	Separation of metals and halogens, breaking down compounds and catalyzing bonds; and nonliving and living materials.	[25,139]
Get, store, or distribute resources	Capture, absorb, or filter. Store, distribute, expel	Organisms, solids, liquids, gases, energy, and chemical entities.	[25,52,144,182]

**Table 6:** Other tools for the application of biomimetics.

Type	Description	Source
Database	ZQ Journal—It shows the synergy between science and biologically inspired design, using case studies, news, and articles relevant to this topic.	[204]
	Global Design Challenge—Offers an annual global bioinspired solution challenge in the contest mode. The annual files can be consulted on this page.	[205]
	ABM HYDRO—Research team on numerical and experimental marine hydrodynamics focused on innovative biomimetic solutions that improve and enable advanced marine operations.	[206]
	Nanophotonics Centre—Research group that studies the optical biomimetics of plants and insects in search of photonic effects.	[207]
	Maxwell Centre—Microbial biophysics for biotechnology and biomimetics.	[208]
Material selectors	Material Pathways—It is part of the research group at the Kolding School of Design’s Sustainability and Design Laboratory. As a result, sustainable approach cards have been designed that can function as a source of inspiration, as ways to mediate knowledge and values in multidisciplinary teams, or as ways to reflect and create analytical awareness.	[209]
	Biomimicry Toolbox—It is a biomimicry manual focused on the “challenge to biology” approach to addressing biomimicry.	[210]
Companies/organizations	Biomimicry 3.8—It is the world’s leading bioinspired consulting firm that offers consulting on biological intelligence, professional training, and inspirational speaking.	[211]
	International Society of Bionic Engineering—The main aim is to bring people together from different disciplines and nations in bionic science, to raise discussions, to create joint strategies and to bring forward the education of the next generations.	[212]
Scientific Journals	Biomimetics—It is an international, peer-reviewed, open-access journal on biomimicry and bionics, published monthly online by MDPI.	[213]
	Journal of Biomimetics, Biomaterials and Biomedical Engineering—Its scope covers the fields of biocompatible materials, biomedical engineering, and biomimetics (descriptions of subjects are given following Medical Subject Headings MeSH).	[214]
Others	Biomimicry DesignLens—Summary of the basic tools of Biomimicry 3.8. It includes design guidelines depending the start point: “from the challenge to biology” or “from biology to design”.	[215]

**Table 7:** Definition and biologization of the design challenge.

Concept	Description
Design question	How can we avoid biofouling on a boat?
Functions	Avoid biofouling, protect, maintain physical integrity.
Context	Marine environment, humid environment, saline environment.
Biologized questions	How does nature… prevent biofouling in humid environments? … protect itself in salty environments? … maintain its physical integrity in the sea?

**Table 8:** Biological strategies and models.

Biological Strategy	Biological Model
Scales protect the skin: cartilaginous fish.	The skin of cartilaginous fish is protected by a protective layer of abrasive placoid scales, called denticles.
The skin influences biofouling: the shark.	Rapidly flowing water near the surface of the skin would reduce the time microorganisms have to settle on the surface and help eliminate those that do settle. Another hypothesis is that the microscopic shape of the shark scales and the topography of their surface prevent the settlement of microorganisms.
Rough surfaces resist biofouling: common mussel, Mediterranean mussel.	The topography of the shell surface consists of a repeating pattern of waves ~1–2 μm wide and ~1.5 μm high. Researchers studying various shell surfaces and their microtopographies found that the “waviness” (overall texture) of the surface correlates with both strength and scale release.
Skin resists microorganisms: pilot whale.	The skin of pilot whales resists microorganisms through microscopic pores and nanoridges, surrounded by a secreted enzymatic gel that denatures proteins and carbohydrates.
Stony corals have microstructures on their surface that prevent biofouling.	Corals have several antifouling strategies. The first is a bioactive antifouling of natural origin. The second is a low surface energy, which decreases the adhesion force to the surface, preventing organisms from adhering. The third is the shedding effect, in which they use a slippery slime to “remove” attached organisms. The fourth is the use of soft external tentacles that prevent organisms from adhering to their surface. And finally, fluorescent pigments are used to absorb harmful UV rays.

**Table 9:** The 40 principles of inventiveness.

Nº	Principle	Nº	Principle
1	Segmentation	21	Rushing through
2	Extraction	22	Convert harm into benefit
3	Local quality	23	Feedback
4	Asymmetry	24	Mediator
5	Consolidation	25	Self-service
6	Universality	26	Copying
7	Nesting	27	Dispose
8	Counterweight	28	Replace of the mechanical system
9	Prior Counteraction	29	Pneumatic or hydraulic constructions
10	Prior action	30	Flexible membranes or thin films
11	Cushon in Advance	31	Porous material
12	Equipotentiality	32	Change the color
13	Do it in reverse.	33	Homogeneity
14	Spheroidality	34	Rejecting and regenerating parts
15	Dynamicity	35	Transformation of properties
16	Partial action	36	Phase transition
17	Transition into a new dimension	37	Thermal expansion
18	Mechanical vibration	38	Accelerated oxidation
19	Periodic action	39	Inert environment
20	Continuity of useful action	40	Composite materials

**Table 10:** Parameters corresponding to BioTRIZ fields.

Fields	Parameters
Substance	Weight, Loss of substance, Amount of substance
Structure	Stability, Complexity, Durability/Robustness/Life
Space	Length, Area, Volume, Shape
Time	Speed, Productivity/Reproduction, Duration of Action
Energy	Force, Stress/Pressure, Strength, Temperature, Illumination Intensity/Brightness, Energy/Power, Function Efficiency, Noise
Information	Security/Protection/Vulnerability, Harmful Effects by System, Harmful Effects on System, Repairability/Healing, Adaptability, Ability to Detect/Precision, Amount of Information (Memory)

**Table 11:** Application of a case study to the BioTRIZ matrix.

Parameters	Substance	Structure	Time	Space	Energy/Field	Information/ Adaptation
Substance	13, 31, 15, 17, 20, 40	1, 2, 3, 15, 24, 26	15, 19, 27, 29, 30	15, 31, 1, 5, 13	3, 6, 9, 25, 31, 35	3, 25, 26
Structure	1, 10, 15, 19	1, 15, 19, 24, 34	**1, 2, 4**	10	1, 2, 4	1, 3, 4, 15, 19, 24, 25, 35
Time	1, 3, 15, 20, 25, 38	1, 2, 3, 4, 6, 15, 17, 19	2, 3, 11, 20, 26	1, 2, 3, 4, 7, 38	3, 9, 15, 20, 22, 25	1, 2, 3, 10, 19, 23
Space	3, 14, 15, 25	2, 3, 4, 5, 10, 15, 19	1, 19, 29	4, 5, 14, 17, 36	1, 3, 4, 15, 19	3, 15, 21, 24
Energy/Field	1, 3, 13, 14, 17, 25, 31	1, 3, 5, 6, 25, 35, 36, 40	3, 10, 23, 25, 35	1, 3, 4, 15, 25	3, 5, 9, 22, 25, 32, 37	1, 3, 4, 15, 16, 25
Information/ Adaptation	1, 6, 22	1, 3, 6, 18, 22, 24, 32, 34, 40	2, 3, 9, 17, 22	3, 20, 22, 25, 33	1, 3, 6, 22, 32	3, 10, 16, 23, 25

**Table 12:** Multicriteria analysis of biomimetic methodologies.

Indicator	Design Spiral	BioTRIZ	AskNature	DANE 2.0	Idea-Inspire
Accessibility of the tool	High	Medium	High	Low	Low
Implementation time	High	Low	Medium	No data	No data
Technical barrier	No	No	No	Yes	Yes
Gap between theory and practice	No	No	No	Yes	Yes
Integration with existing design processes	High	High	High	High	High
Accessibility and usability	Yes	Yes	Yes	No	No
Knowledge level required	Low	High	Low	High	High

## Data Availability

The original contributions presented in the study are included in the article/Supplementary Materials, further inquiries can be directed to the corresponding author/s.

## Supplementary Materials

The following supporting information can be downloaded at: https://www.mdpi.com/article/10.3390/biomimetics9090523/s1. Table S1 includes an extensive biomimicry taxonomy of Table 5, listing specific functions along with application examples.
