# Supplementary material for: Innovation Inspired by Nature: Applications of Biomimicry in Engineering Design
Source: Biomimetics (Basel). 2024 Aug 30;9(9):523. doi: 10.3390/biomimetics9090523 (PMC11429577; doi:10.3390/biomimetics9090523)
Supplement: Supplementary file 1 [file biomimetics-09-00523-s001.zip › biomimetics-3139561-supplementary.pdf]

Group	Sub-group	Function	Examples
Move or stay put	Attach	Permanently	[1]
		Temporarily	[2,3]
	Move	In/on solids	[4]
		In/on liquids	[4]
		In/throught gases	[2,4–8]
Protect from physical harm	Protect from living threats	Animals	[9–11]
		Plants	[9–12]
		Fungi	[10–12]
		Microbes	[10–12]
	Protect from non-living threats	Excess liquids	[10,11,13–15]
		Loss of liquids	[10,11,13–15]
		Loss of gases	[10,11,13,14]
		Light	[10,11,13,14]
		Temperature	[10,11,13,14,16]
		Wind	[10,11,13,14]
		Gases	[10,11,13,14]
		Dirt/solids	[10,11,13,14,17]
		Chemicals	[10,11,13,14]
		Fire	[10,11,13,14]
		Ice	[10,11,13,14]
		Nuclear radiation	[10,11,13,14]
	Manage structural forces	Shear	[10,11,13–15,18,19]
		Compression	[10,11,13–15,18,19]
		Thermal shock	[10,11,13–15,18,19]
		Impact	[10,11,13–15,18,19]
		Tension	[10,11,13–15,18,19]
		Turbulance	[10,11,13–15,18,19]
		Mechanical wear	[10,11,13–15,18,19]
		Chemical wear	[10,11,13–15,18,19]
		Creep	[10,11,13–15,18,19]
	Regulate physiol. Processes	Cellular processes	[20]
		Maintain homeostasis	-
		Reproduction or growth	-
	Prevent structural failure	Buckling	[10,11,14,16,19]
		Deformation	[10,11,14,16,19]
		Fatigue	[10,11,14,16,19]
		Melting	[10,11,14,16,19]
		Fractue/rupture	[10,11,14,16,19]
	Coordinate	Coordinate by self-organization	[9,21]
		Activities	[9,21]
		Systems	[9,21]
Maintain community	Cooperate	Within the same species	[21,22]
		Cooperate/compete between different species	[21,22]
		Within a (eco)system	[21,22]
		Cooperate/compete between (eco)systems	[21,22]
	Provide ecosystem services	Manage disturbance in a community	-
		Regulate hydrological flows	[15]
		Polinate	-

		Generate soil/renew fertility	-
		Detoxification/purification of air/water/waste	[17,20]
		Control erosion and sediment	[15]
		Regulate water storage	[15]
		Cycle nutrients	[20]
		Regulate atmospheric composition	-
		Regulate climate	[20]
		Disperse seeds	
		Maintain biodiversity	[20]
		Biological control of populations, pests, diseases	[17,23]
Modify	Modify physical state	Size/shape/mass/volume	[24]
		Pressure	[24]
		Density	[24]
		Phase	[24]
		Buoyancy	[24]
		Light/color	[13,24,25]
		Material characteristics	[13,24,25]
		Speed	[24]
		Position	[24]
	Modify chemical/electrical state	Energy state	[4]
		Free radical reactivity	[26]
		Concentration	-
		Chemical potential	[26]
		Reactivity with water	-
		Oxidation state	[26]
		Electric charge	-
		Conductivity	[13,26]
		Surface tension	[13]
		Ph	[135]
		Solubility	-
		Electron transport	-
		Chemically generate flow of electrons	[135]
	Adapt/optimize	Adapt genotype	[27,51]
		Adapt phenotype	[27,51]
		Coevolve	[51]
		Adapt behaviors	[51]
		Optimize space/materials	[27,37,51,127,181]
	Transform/convert energy	Electrical energy	[28]
		Magnetic energy	-
		Chemical energy	[28]
		Mechanical energy	-
		Thermal energy	[28]
		Radiant energy (light)	[28]
Make	Reproduce	Self-replicate	-
	Physically assemble	Structure	[37,135,140]
	Chemically assemble	Polymers	[20,135,140,186]
		Metal-based compounds	[20,135,140]
		Molecular devices	[20,135,140]
		Specific stereoisomers	[20,135,140]
		Mineral crystals	[20,135,140]
		Inorganic compounds	[20,135,140,186]
		Organic compounds	[20,135,140,186]
		Attach a functional group	[20,135,140,186]
		Detach a functional group	[20,135,140,186]
		Catalyze making of bonds	[20,135,140]

		On demand	[20,135,140]
Process information	Navigate	Through air	[128]
		Through liquid	[128]
		Over land	[128]
		Through solids	[128]
	Send signals	Light (visible spectrum)	[28]
		Light (non-visible spectrum)	[28]
		Sound	-
		Tacticle	-
		Chemical (odor, taste, etc)	-
		Vibratory	-
		Electrical/magnetic	-
	Process signals	Differentiate signal from noise	[33,115,119]
		Transduce/convert signals	[33,115,119]
		Respond to signals	[27,33,115,119]
	Sense signals/environmental cues	Light (visible spectrum)	[28]
		Light (non-visible spectrum)	[28]
		Electricity/magnetism	-
		Disease	-
		Touch and mechanical forces	-
		Chemicals (odor, taste, etc.)	[20]
		Atmospheric conditions	-
		Sound and other vibrations	-
		Temperature	-
		Motion	[92]
		Pain	-
		Body awareness	-
		Balance/orientation	-
		Shape and pattern	[27]
		Time and day length	-
	Compute/learn/[en/de]code		-
Break down	Chemically break down	Cleave heavy metals from organic compounds	[20,135]
		Cleave halogens from organic compounds	[20,135]
		Inorganic compounds	[20,135]
		Polymers	[20,135]
		Organic compounds	[20,135]
		Catalyze making of bonds	[20,135]
	Physically break down	Non-living materials	[135]
		Living materials	[135]
Get, store or distribute resources	Capture, absorb, or filter	Organisms	[20,140]
		Solids	[20,140,181]
		Liquids	[20,140]
		Gasses	[20,140]
		Energy	[20,28,51,140]
		Chemical entities	[140]
	Store	Solids	[140,181]
		Liquids	[140,181]
		Gases	[140]
		Energy	[51,140]
		Chemical entities	[20,140]
	Distribute	Solids	[140]
		Liquids	[140]
		Gases	[140]
		Energy	[51,140]
	Expel	Solids	-

		Liquids	-
		Gases	

1. Tang, Z.; Wang, Y.; Podsiadlo, P.; Kotov, N. Biomedical Applications of Layer-by-layer Assembly: From Biomimetics to Tissue Engineering. *Wiley Online Library* **2006**, *18*, 3203–3224, doi:10.1002/adma.200600113.
2. Rolf, M.; Neumann, K.; Queißer, J.F.; Reinhart, R.F.; Nordmann, A.; Steil, J.J. A Multi-Level Control Architecture for the Bionic Handling Assistant. *Taylor & Francis* **2015**, *00*, 1–15.
3. León, H.; Gama-Campillo, A.; Catillo, E.; Ortiz, H. Innovación En El Diseño Biomimético de Robots Available online: https://scholar.google.es/scholar?hl=es&as_sdt=0%2C5&q=Innovaci%C3%B3n+en+el+Dise%C3%B1o+Biomim%C3%A9tico+de+robots+Hexapodos+y+Cuadrupedos.&btnG= (accessed on 17 May 2023).
4. Carpi, F.; Erb, R.; Jeronimidis, G. Special Section on Biomimetics of Movement. *iopscience.iop.org* **2011**.
5. Billard, A.; Matarić, M. Learning Human Arm Movements by Imitation: Evaluation of a Biologically Inspired Connectionist Architecture. *Elsevier* **2001**, *941*, 1–16.
6. Low, K.; Hu, T.; Mohammed, S. Perspectives on Biologically Inspired Hybrid and Multi-Modal Locomotion. *iopscience.iop.org* **2015**.
7. Siddall, R.; Biomimetics, B.&; Low, K.H.; Hu, T.; Mohammed, S. Launching the AquaMAV: Bioinspired Design for Aerial–Aquatic Robotic Platforms. *iopscience.iop.org* **2014**, doi:10.1088/1748-3182/9/3/031001.
8. Bogue, R. Bioinspired Designs Impart Robots with Unique Capabilities. *Industrial Robot* **2019**, *46*, 561–567, doi:10.1108/IR-05-2019-0100/FULL/HTML.
9. Ren, L.; Li, B.; Wei, G.; Wang, K.; Song, Z.; Wei, Y.; Iscience, L.R. Biology and Bioinspiration of Soft Robotics: Actuation, Sensing, and System Integration. *Elsevier* **2021**.
10. Gebeshuber, I.C.; Stachelberger, H.; Ganji, A.; Fu, D.C.; Yunas, J.; Majlis, B.Y. Exploring the Innovational Potential of Biomimetics for Novel 3D MEMS. **2009**, doi:10.4028/www.scientific.net/AMR.74.265.
11. Cadman, J.; Zhou, S.; Chen, Y.; Engineering, Q.L.-J. of B.; 2012, undefined Cuttlebone: Characterisation, Application and Development of Biomimetic Materials. *Springer* **2012**, *9*, 367–376, doi:10.1016/S1672-6529(11)60132-7.
12. Yan, H.; Wu, Q.; Yu, C.; Zhao, T.; Liu, M. Recent Progress of Biomimetic Antifouling Surfaces in Marine. *Adv Mater Interfaces* **2020**, *7*, doi:10.1002/ADMI.202000966.
13. Xia, F.; Jiang, L. Bio-inspired, Smart, Multiscale Interfacial Materials. *Wiley Online Library* **2008**, *20*, 2842–2858, doi:10.1002/adma.200800836.
14. Rezaei, D.; Zare, M. Plants in Arid Climate as a Pattern for Bionic Architecture in Behavioral Viewpoint Available online: https://www.researchgate.net/publication/316352839_Plants_in_arid_climate_as_a_pattern_for_bionic_architecture_in_behavioral_viewpoint (accessed on 15 May 2023).

15. Kenny, J.; Kumar, A.; Kiran, C. Exploring a Biomimicry Based Approach and Key Barriers to Evolving Water Infrastructure Systems Available online: https://www.researchgate.net/publication/281850099_Exploring_a_biomimicry_based_approach_and_key_barriers_to_evolving_water_infrastructure_systems (accessed on 15 May 2023).

16. Milwich, M.; Speck, T.; Speck, O.; Stegmaier, T.; Planck, H. Biomimetics and Technical Textiles: Solving Engineering Problems with the Help of Nature's Wisdom. *Wiley Online Library* **2006**, *93*, 1455–1465, doi:10.3732/ajb.93.10.1455.

17. Schroeder, A.; Marshall, L.; Trease, B. Development of Helical, Fish-Inspired Cross-Step Filter for Collecting Harmful Algae. *iopscience.iop.org* **2019**.

18. Qi Wang, R. Learn from Reptiles: A Model of Bionic Architecture | BioBuild Program Available online: https://biobuild.mlsoc.vt.edu/blog/learn-reptiles-model-bionic-architecture (accessed on 15 May 2023).

19. Biology, S.R. Search the Structures of Bionic Principles for Designing Tensegrity Structures. *aensiweb.net* **2014**, *8*, 714–721.

20. García-Serna, J.; Pérez-Barrigón, L.; Cocero, M. New Trends for Design towards Sustainability in Chemical Engineering: Green Engineering. *Elsevier* **2007**.

21. Krzemińska, A.E.; Zaręba, A.D.; Dzikowska, A.; Jarosz, K.R. Cities of the Future—Bionic Systems of New Urban Environment. *Environmental Science and Pollution Research* **2019**, *26*, 8362–8370, doi:10.1007/S11356-017-0885-2.

22. Pfeifer, R.; Lungarella, M.; Iida, F. Self-Organization, Embodiment, and Biologically Inspired Robotics. *Science (1979)* **2007**, *318*, 1088–1093, doi:10.1126/SCIENCE.1145803.

23. Solga, A.; Cerman, Z.; Striffler, B.F.; Spaeth, M.; Barthlott, W. The Dream of Staying Clean: Lotus and Biomimetic Surfaces. *iopscience.iop.org* **2007**, *2*, 126–134, doi:10.1088/1748-3182/2/4/S02.

24. Clegg, J.; Wagner, A.; Shin, S.; … S.H. Modular Fabrication of Intelligent Material-Tissue Interfaces for Bioinspired and Biomimetic Devices. *Elsevier* **2019**.

25. Saito, A. Material Design and Structural Color Inspired by Biomimetic Approach. *Sci Technol Adv Mater* **2011**, *12*, 064709, doi:10.1088/1468-6996/12/6/064709.

26. Li, B.; Haynie, D. Multilayer Biomimetics: Reversible Covalent Stabilization of a Nanostructured Biofilm. *ACS Publications* **2004**, *5*, 1667–1670, doi:10.1021/bm0496155.

27. Shah, D.; Yang, B.; Kriegman, S.; Levin, M.; Bongard, J.; Kramer-Bottiglio, R. Shape Changing Robots: Bioinspiration, Simulation, and Physical Realization. *Wiley Online Library* **2021**, *33*, doi:10.1002/adma.202002882.

28. RANT, D.; PAVKO-ČUDEN, A. SELF-FOLDABLE KNITTED BAGS FOR HOUSEHOLD STORAGE OF BAKERY PRODUCTS BASED ON BIOMIMETICS. *bib.irb.hr* **2018**.

29. Malfaz, M.; Castro, Á.; Barber, R.; Salichs, M.A. A Biologically Inspired Architecture for an Autonomous and Social Robot. *IEEE Trans Auton Ment Dev* **2011**, *3*, 232–246, doi:10.1109/TAMD.2011.2112766.

30. Jacobsen, R.; Zhang, Q.; Toftegaard, T. Bioinspired Principles for Large-Scale Networked Sensor Systems: An Overview. *mdpi.com* **2011**, *11*, 4137–4151, doi:10.3390/s110404137.

31. Valle, M. Bioinspired Sensor Systems. *Sensors 2011, Vol. 11, Pages 10180-10186* **2011**, *11*, 10180–10186, doi:10.3390/S111110180.
